# Monitoring mouse papillomavirus-associated cancer development using longitudinal Pap smear screening

**DOI:** 10.1128/mbio.01420-24

**Published:** 2024-07-16

**Authors:** Hannah M. Atkins, Aysegul Aksakal Uslu, Jingwei J. Li, Debra A. Shearer, Sarah A. Brendle, Chen Han, Michael Kozak, Paul Lopez, Deesha Nayar, Karla K. Balogh, Catherine Abendroth, Jean Copper, Keith C. Cheng, Neil D. Christensen, Yusheng Zhu, Stefanie Avril, Adam D. Burgener, Thomas T. Murooka, Jiafen Hu

**Affiliations:** 1 Department of Pathology and Laboratory Medicine, Division of Comparative Medicine, The University of North Carolina, Chapel Hill, North Carolina, USA; 2 The Jake Gittlen Laboratories for Cancer Research, Pennsylvania State University College of Medicine, Hershey, Pennsylvania, USA; 3 Department of Pathology and laboratory medicine, Pennsylvania State University College of Medicine, Hershey, Pennsylvania, USA; 4 TEM facility, Pennsylvania State University College of Medicine, Hershey, Pennsylvania, USA; 5 Department of Immunology, The University of Manitoba, Winnipeg, Manitoba, Canada; 6 Department of Biochemistry and Molecular Biology, Pennsylvania State University College of Medicine, Hershey, Pennsylvania, USA; 7 Department of Microbiology and immunology, Pennsylvania State University College of Medicine, Hershey, Pennsylvania, USA; 8 Department of Pathology, School of Medicine, Case Western Reserve University, Cleveland, Ohio, USA; 9 Center for Global Health and Diseases, University of Manitoba, Winnipeg, Canada; 10 Department of Obstetrics, Gynecology, and Reproductive Sciences, University of Manitoba, Winnipeg, Canada; 11 Department of Medicine, Unit of Infectious Diseases, Center for Molecular Medicine, Karolinska Institutet, Solna, Stockholm, Sweden; Institute of Microbiology and Immunology, Faculty of Medicine, University of Ljubljana, Ljubljana, Slovenia

**Keywords:** the mouse papillomavirus (MmuPV1), lower genital infection, Pap smear, LSIL, HSIL, squamous cell carcinoma, longitudinal, cytology, *in situ* hybridization, RNAscope, transmission electron microscope (TEM), 2014 Bethesda system, qPCR, mouse model, immunohistochemistry (IHC), viral copy number

## Abstract

A substantial percentage of the population remains at risk for cervical cancer due to pre-existing human papillomavirus (HPV) infections, despite prophylactic vaccines. Early diagnosis and treatment are crucial for better disease outcomes. The development of new treatments heavily relies on suitable preclinical model systems. Recently, we established a mouse papillomavirus (MmuPV1) model that is relevant to HPV genital pathogenesis. In the current study, we validated the use of Papanicolaou (Pap) smears, a valuable early diagnostic tool for detecting HPV cervical cancer, to monitor disease progression in the MmuPV1 mouse model. Biweekly cervicovaginal swabs were collected from the MmuPV1-infected mice for viral DNA quantitation and cytology assessment. The Pap smear slides were evaluated for signs of epithelial cell abnormalities using the 2014 Bethesda system criteria. Tissues from the infected mice were harvested at various times post-viral infection for additional histological and virological assays. Over time, increased viral replication was consistent with higher levels of viral DNA, and it coincided with an uptick in epithelial cell abnormalities with higher severity scores noted as early as 10 weeks after viral infection. The cytological results also correlated with the histological evaluation of tissues harvested simultaneously. Both immunocompromised and immunocompetent mice with squamous cell carcinoma (SCC) cytology also developed vaginal SCCs. Notably, samples from the MmuPV1-infected mice exhibited similar cellular abnormalities compared to the corresponding human samples at similar disease stages. Hence, Pap smear screening proves to be an effective tool for the longitudinal monitoring of disease progression in the MmuPV1 mouse model.

**IMPORTANCE:**

Papanicolaou (Pap) smear has saved millions of women's lives as a valuable early screening tool for detecting human papillomavirus (HPV) cervical precancers and cancer. However, more than 200,000 women in the United States alone remain at risk for cervical cancer due to pre-existing HPV infection-induced precancers, as there are currently no effective treatments for HPV-associated precancers and cancers other than invasive procedures including a loop electrosurgical excision procedure (LEEP) to remove abnormal tissues. In the current study, we validated the use of Pap smears to monitor disease progression in our recently established mouse papillomavirus model. To the best of our knowledge, this is the first study that provides compelling evidence of applying Pap smears from cervicovaginal swabs to monitor disease progression in mice. This HPV-relevant cytology assay will enable us to develop and test novel antiviral and anti-tumor therapies using this model to eliminate HPV-associated diseases and cancers.

## INTRODUCTION

Human papillomavirus infection is one of the most common sexually transmitted diseases (1). HPV is the causative factor for almost all cervical cancers, 70% of vaginal and oropharyngeal cancers, and 90% of anal cancers (2–5). In 2020, as the fourth most common cancer, an estimated 604,127 cervical cancer cases and 341,831 deaths were reported globally (6). Worldwide, two in three women aged 30–49 years have never been screened for cervical cancer (7). More than three million cases of HPV-associated cervical intraepithelial neoplasia (CIN) were detected in the United States in 2019. Four prophylactic virus-like particle (VLP)-based vaccines have shown strong protection against up to nine high-risk HPV types included in the vaccines, even with the new single-dose regimen to counteract the limited vaccine supply (8, 9). However, these vaccines cannot protect against HPV infections from types not covered by the vaccine, and they do not exhibit therapeutic effects for pre-existing infections (10). Considering that the vaccine uptake rates for the full doses in eligible populations remain around 50% in the United States and even lower in low-income countries (11), HPV-associated infections and cancers will continue to be a significant public health problem (11, 12). Although excisional treatment of pre-invasive disease is effective, lesions often reoccur (2). Therefore, innovative therapeutic approaches to manage HPV-associated cancers are essential for the millions of people who contract HPV infections each year. A better understanding of the mechanisms behind HPV pathogenesis is essential for the development of these HPV-associated cancer treatments (13).

Early diagnosis is critical for the control of HPV-associated disease progression (14, 15). The Papanicolaou test, also called Pap smear or Pap test, has been used successfully for cervical screening since 1943 (15). This test includes collecting cells from the transition zone area of the cervix and detecting potentially precancerous and cancerous processes (16, 17). Detecting pre-invasive lesions has saved millions of lives (15). One critical challenge for studying HPV *in vivo* is that HPV infections are species-specific. Thus, no preclinical model system can be used to study HPV infection directly (18). Recently, we and others established a mouse papillomavirus (MmuPV1) model that displays relevance to HPV infections in the anogenital tract and oropharynx (19–31). Our published and unpublished data strongly support that the infection course of MmuPV1 mimics HPV infections in the human lower genital tract with increased detection of viral signals in the cervicovaginal specimens and disease progression from low-grade to high-grade dysplasia over time (21, 22, 32). Advanced squamous cell carcinomas (SCC) in the vaginal tissues have been demonstrated in different mouse strains, including immunocompetent mouse strains (FVB and NU/J heterozygotes), immunocompromised (athymic nude mice), and immunogenetically modified mice (19–21). We also developed noninvasive methods for longitudinally monitoring viral activities by quantitating viral DNA copies *via* qPCR analysis (21, 22, 32, 33). All these preliminary studies led us to postulate that a Pap smear method might be appropriate for monitoring disease progression in the mouse HPV model.

The current study took advantage of our longitudinal monitored MmuPV1 lower genital infection model (34), the well-established human Pap smear (16), and the 2014 Bethesda system (16, 17, 35) to demonstrate that Pap smear is a valuable method for monitoring disease progression in the mouse PV model. We included two clinically relevant immunogenetically modified mouse strains, NU/J heterozygous (Foxn1^nu/+^) and Rag1ko mice, to represent immunocompetent and immunocompromised backgrounds, respectively. Transcription factor forkhead box N1 (Foxn1) is the master regulatory gene of thymic epithelium development and function (36, 37). In heterozygous (Foxn1^nu/+^) human patients, the Foxn1 gene dosage is more critical during embryonic and early post-natal life, with an increased risk of complication due to increased susceptibility to pathogens. However, these patients showed normal T-lymphocyte function as adults (31). Likewise, after 3 weeks of age, NU/J heterozygous (Foxn1^nu/+^) mice also showed comparable thymocyte counts to wild type (Foxn1^+/+^) (31). Therefore, 4- to 6-week-old NU/J heterozygous (Foxn1^nu/+^) mice used in the current study are considered immunocompetent. These mice were tested in our previous study and proceeded to develop advanced diseases, including carcinoma *in situ* in the lower genital infected tissues (21). In addition to NU/J heterozygous (Foxn1^nu/+^) mice, a second mouse strain, recombination-activating genes 1 (Rag1) knockout mice, was included in the current study as the positive control for viral infection (27, 38–40). Rag1 and Rag2 proteins play an essential role in adaptive immunity by initiating V(D)J recombination (41–43). Patients without Rag1 or Rag2 display lymphocyte development arrested, resulting in T- and B-cell deficiency (41–43). Similarly, Rag1 and Rag2 knockout mice have no T- and B-cell responses and are used to study different diseases (41, 44–46). Rag1ko mice are susceptible to MmuPV1 infections in both cutaneous and mucosal tissues and have been used as the positive control for MmuPV1 infection in previous studies (27, 38, 40).

We followed vaginally infected mice longitudinally from benign to advanced disease stages, including squamous cell carcinoma in Rag1ko and NU/J heterozygous (Foxn1^nu/+^) mice for up to 32 weeks. Cervicovaginal swabs were periodically collected for viral DNA analysis *via* qPCR (32) and for cytological assays *via* Pap smears. At the termination of the experiments, infected tissues from these tested mice were further analyzed using additional *in situ* assays to confirm viral presence (21) and disease stages by histology. To further validate our observations and their relevance to HPV infections and associated cancer, we compared our results with corresponding human Pap smear samples and cervical interepithelial neoplasia tissues. The demonstrated surprising similarities in cytology and histology stages between diseased human and mouse cervicovaginal lavage samples will lay a solid foundation for us to study mechanisms of HPV disease progression and test novel anti-viral and anti-tumor compounds in our mouse PV model.

## MATERIALS AND METHODS

### Mice and MmuPV1 infection

All mouse work was approved by the Institutional Animal Care and Use Committee of Pennsylvania State University’s College of Medicine (COM), and all methods were performed in accordance with guidelines and regulations. The mice were housed (2–5 mice/cage) in sterile cages within sterile filter hoods in the COM BL2 animal core facility. Immunocompromised mouse strains, including Rag1ko (B6.129S7-Rag1tm1Mom/J, 002216, the Jackson laboratory), athymic nude mice (Hsd:NU, Envigo) (21), and immunocompetent mouse strain NU/J heterozygous mice (002019, the Jackson laboratory) (21) were used for the study.

Infectious MmuPV1 virions were isolated from lesions on the tails of athymic nude mice from our previous study (24). In brief, lesions scraped from the tails of the Hsd:NU athymic mice were homogenized in sterile phosphate-buffered saline (1 × PBS) using a polytron homogenizer (Brinkman PT10-35) at the highest speed for 3 minutes while chilling in an ice bath. The homogenate was spun at 10,000 rpm and the supernatant was mixed with glycerol (1:1) and decanted into Eppendorf tubes for long-term storage at −80°C. The viral DNA of the stock was quantitated by extracting the DNA from 5 µL of this stock using qPCR. For standard infection, 10 µL of MmuPV1 virus stock (1 × 10^8^ viral DNA equivalents) was used for infection in the lower genital tract (47).

To increase the infectivity and generate robust results, all female mice were treated with 3 mg of medroxyprogesterone acetate (Depo-Provera, DMPA) subcutaneously 3 days before the viral infection as previously described (32). The day before infection, the mice were anesthetized, and the lower genital tracts were pre-wounded with Doctors’ Brush Picks coated with 4% nonoxynol-9 gel [Conceptrol^(R)^]. Twenty-four hours after pre-wounding, the mice were again anesthetized and challenged with infectious MmuPV1 virus (1 × 10^9^ viral DNA equivalents) using a filtered pipette tip in the lower genital tract as described in our previous studies (47). For the titration study in NU/J heterozygous mice, four groups of mice (*N* = 5/group) were infected with the same volume of viral suspension between 1 × 10^5^ and 1 × 10^8^ viral DNA equivalents in sterile 1 × PBS.

### Cervicovaginal swab for DNA extraction and qPCR analysis

Biweekly swabs from the lower genital tract were monitored for viral DNA by qPCR (32). Vaginal swabs (Puritan PurFlock Ultra; Puritan Diagnostics, LLC) soaked in 25 µL of sterile 0.9% NaCl were introduced into the vaginal canals and then stored at –20°C before processing for viral DNA quantification. Viral DNA was extracted (DNeasy Blood & Tissue Kit, 69506), and the qPCRs were run in an Agilent qPCR machine as described previously (24). The primer pairs (5′GGTTGCGTCGGAGAACATATAA 3′ and 5′ CTAAAGCTAACCTGCCACATATC 3′) for E2 amplification were used together with the probe (5′ 6-FAM-TGCCCTTTCA/ZEN/GTGGGTTGAGGACAG 3′-IBFQ). Each reaction contained 9 µL ultrapure water, 5 pmol of each primer, 9 µL Brilliant III qPCR Master Mix (Agilent), and 2 µL DNA template. The PCR conditions were initial denaturation at 95°C for 3 minutes, then 40 cycles at 95°C for 5 seconds, and 60°C for 10 seconds on an Agilent AriaMx. Viral copy numbers were converted into an equivalent DNA load: 1 ng viral DNA = 1.2 × 10^8^ copies (http://cels.uri.edu/gsc/cndna.html). All samples were assessed in duplicates or triplicates (21). Viral titers were calculated according to the standard curve (32).

### Viral RNA detection

RNA samples were extracted from mouse tissues that were flash-frozen in liquid nitrogen and stored at −80°C or liquid nitrogen. Approximately 20 mg of tissue was put into 1 mL TRIzol and processed for 5 minutes through a homogenizer (24). Following the standard Thermo Fisher protocols for TRIzol, the clear supernatant was collected, and 0.2 mL chloroform was added per 1 mL TRIzol solution. The mixtures were centrifuged, and the clear supernatant was retrieved. RNA was quantified using a nanodrop Spectrophotometer (NanoDrop Technologies; ND-1000), and 1 µg RNA was used for the reverse-transcriptase reaction using the SuperScript III RT Kit (Thermo Fisher Scientific; 18080044). The copy numbers were based on 200 ng RNA from each sample (Viral RNA E1^E4 transcripts were quantified using primers 5′-TAGCTTTGTCTGCCCGCACT-3′ and 5′-GTCAGTGGTGTCGGTGGGAA-3′ and probe 5’FAM-CGGCCCGAAGACAACACCGCCACG-3′TAMRA. RNA (0.2 µg) was mixed with 0.25 µg/µL random primers, 10 mM dNTP mix, and nuclease-free H_2_O for a total of 13 µL. This sample was then incubated at 65°C for 5 minutes. A mix of diluted 1 × first-strand buffer, 0.1 M dithiothreitol, 40 U/ µL RNase, and 200 U SuperScript III was added to the nucleotide mix. The sample was then incubated in a thermocycler. The program consisted of 5 minutes at 25°C, 1 hour at 50°C, and 15 minutes at 70°C. Following PCR, cDNA samples were diluted 1:9 using Ultra-Pure DNase/RNase-Free Distilled Water. Of the 1:9 dilution, 2 µL was used in the 20 µL quantitative PCR (qPCR) reaction purchased from Agilent.

### Cytology and histology assessment and interpretation

**Fig 1:**
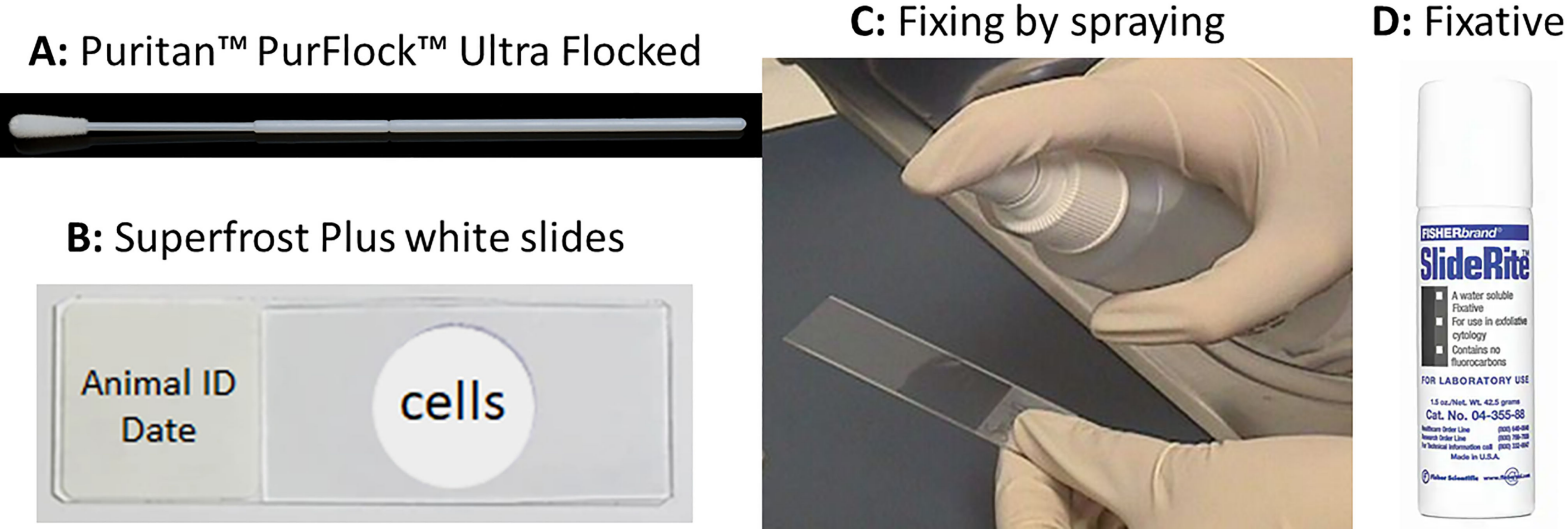
Pap smear slide preparation using a conventional swab. Single-used swabs (A, Puritan PurFlock Ultra Flocked Swabs) were dipped into sterile 0.9% NaCl and inserted into the vaginal tract of infected mice. The cells on the swabs were spread onto a superfrost white slide (B) by rotating the swabs on the slide in a circle manner. The cells will be fixed by spraying (C) the SlideRight cytology fixative (D, fishersci) from 6 inches above. The fixed slides were stored in a slide box at 4°C before staining with the standard Pap smear protocol.

Cervicovaginal swab samples were collected from noninfected control and MmuPV1-infected animals at different times post-viral infection. Cells on the swabs were spread by rolling on labeled slides in a circle motion (22) and fixed by spraying the SlideRight cytology fixative (Fishersci) from 6 inches above the slide as shown in Fig. 1. The fixed slides were kept at 4°C before Pap smear staining. All cytology slides were automatically stained using an automatic stainer with the standard protocol Pap smear reagent used by human samples (Thermo Scientific/Richard Allen Scientific). In brief, the fixed slides were sequentially hydrated by being submerged in 100%, 95%, and 70% ethanol for 2 minutes each. After washing in ddH_2_O for 1 minute, the slides were stained with Gill’s hematoxylin for 3 minutes. After a 3-minute wash with ddH_2_O, the slides were placed in bluing for 1 minute, ddH_2_O for 1 minute, 70% ethanol for 1 minute, OG-6 for 3 minutes, two changes of 95% ethanol for 2 minutes, EA-50 for 3 minutes, and three changes of 95% ethanol for 2 minutes. Finally, slides were submerged in 100% ethanol for 3 minutes and xylene for 3 minutes before being mounted and cover-slipped with Cytoseal 60. The slides were scanned 7–10 days after staining to ensure the mounting media was completely dry.

The stained slides were scanned digitally at 40× magnification using an Aperio AT2 digital slide scanner at the Aperio Slide Scanning Core at Penn State COM. The digital slides were independently evaluated by one veterinary pathologist and a physician pathologist for signs of epithelial cell abnormalities using the ImageScope x64 software (Table 1). Both pathologists (HMA and AU) were provided with sample IDs without knowledge of infection status or time post-infection before matching the assessments with the animal ID. Modified 2014 Bethesda system criteria comparable to that used for human cytological samples representing grades of intraepithelial neoplasia were used for these slides, considering normal mouse physiology (48): (i) Normal: normal squamous cells with oval-shaped and small nuclei. They are negative for intraepithelial lesion and malignancy; (ii) ASC-US: Atypical squamous cells of uncertain significance with nucleus size 2–2.5 times larger than a normal squamous cell; (iii) LSIL: Low-grade squamous intraepithelial lesion with hyperchromatic raisin shaped nuclei with a clear halo around it or binucleated cells; (iv) ASC-H: Atypical squamous cells with nucleus size 2–2.5 times larger than a normal squamous cell, cannot exclude HSIL; (E) HSIL: High-grade squamous intraepithelial lesion (HSIL, hyperchromatic cells with high N-C ratio and forming sheets), encompassing cervical intraepithelial neoplasia grades 2 and 3; CIN2 and CIN3; and F) SCC: squamous cell carcinoma (SCC, keratinizing with marked pleomorphism of cell size and shape, arrow; the presence of tadpole cells with dense orangeophilic cytoplasm, arrow; spindle-shaped cells, and inflammation and necrotic debris on the background).

**TABLE 1:** The final diagnosis of Pap smear slides by the two pathologists (HMA and AU)

Slide ID	HMA	Gul	Slide ID	HMA	Gul
31592	IELM Neg	IELM Neg	31096	HSIL	HSIL
31593	LSIL	LSIL	31099	HSIL	HSIL
31594	ASC-H	ASC-H	31140	SCC	SCC
31595	ASC-US	ASC-US	31141	SCC	SCC
31596	ASC-H	LSIL	31142	SCC	SCC
31597	ASC-US	ASC-US	31144	LSIL	LSIL
31598	IELM Neg	IELM Neg	31145	ASC-H	ASC-H
31600	IELM Neg	IELM Neg	31146	ASC-H	ASC-H
31601	IELM Neg	IELM Neg	31147	HSIL	HSIL
31602	ASC-US	ASC-US	31149	HSIL	HSIL
31603	HSIL	HSIL	31151	HSIL	HSIL
31604	LSIL	LSIL	31152	HSIL	HSIL
31605	ASC-US	ASC-US	31157	SCC	SCC
31607	IELM Neg	IELM Neg	31158	ASC-H	ASC-H
31608	LSIL	LSIL	31159	SCC	SCC
31610	LSIL	LSIL	31160	SCC	SCC
31611	IELM Neg	IELM Neg	31161	SCC	SCC
31612	LSIL	LSIL	31162	HSIL	HSIL
31613	LSIL	LSIL	31163	ASC-H	ASC-H
31614	LSIL	LSIL	31164	HSIL	HSIL
31615	ASC-US	ASC-US	31165	ASC-H	ASC-H
31616	ASC-US	ASC-US	31167	SCC	SCC
31617	ASC-H	ASC-H	31168	HSIL	HSIL
31618	SCC	SCC	31169	HSIL	HSIL

### Anti-MmuPV1 antibody detection by enzyme-linked immunosorbent assays (ELISA)

Mouse sera were collected at the termination of the experiment to assess for antibodies against E4. 0.1–0.5 µg of KLH-conjugated MmuPV1 E4 peptide (PKTTPPRRELFPPTPLTQPP, synthesized by China peptide)/well in 50 µL bicarbonate (pH 9.6) buffer was incubated overnight at 37°C. Coated plates were blocked with PBS/5% dry milk powder for 1 hour at room temperature. The ELISA was conducted as reported previously (24, 27). Each serum sample was diluted 1:100 to 1:100,000 in PBS/5% dry milk and then added to the 96-well plates for 1-hour incubation at RT. Plates underwent five washes with washing buffer [0.05% (vol/vol) Tween 20 in 1 × PBS], before the addition of secondary anti-Ig isotypes (IgG3)-alkaline phosphatase (AP) (Southern Biotech) diluted 1:2,000 in 5% milk/1 × PBS for 1 hour at RT. After five further washes, 100 µL of 1 mg/mL pNPP (para-nitrophenyl phosphate, Sigma), a substrate for AP, was added to each well for color development, and absorbance at 405 nm/450 nm was measured using a Fisher Microplate Reader.

### Western blot analysis for detection of L1 protein

Proteins were extracted from paraffin-embedded vagina tissues using the Qproteome FFPE tissue kit (ID: 37623) according to the manufacturer’s instructions. Protein concentration was determined with a BCA Protein Assay kit (Thermo Fisher Pierce). 15 µg of proteins from vaginal tissues of either Rag1ko or NU/J heterozygous mice (four separate samples) was denatured at 95°C for 5 min, separated by gel electrophoresis using a Novex Wedge Well 4%–20% Tris-Glycine Gel (Thermo Fisher Invitrogen) and transferred to a PVDF membrane (Merck Millipore). MPV.B9 (1:200) or beta-actin (1:200) and Anti-mouse IgG1 HRP (1: 2,000, Thermo Fisher Pierce) were used to probe for L1 in western blot analysis (49). A 10 µg positive L1 VLP sample was used as a positive control. The samples were run and probed as described above. Protein loading controls were confirmed by Coomassie stain (data not shown).

### Immunohistochemistry and *in situ* hybridization analyses of infected tissues

After the termination of the experiment, the animals were euthanized. Half of each reproductive tract, including vaginal tissue of interest, was frozen in liquid nitrogen and the other half was fixed in 10% buffered formalin then paraffin-embedded and sectioned as described previously (22). Regressive hematoxylin and eosin (H &E) staining, *in situ* hybridization (ISH), and immunohistochemistry (IHC) were conducted routinely in the laboratory as described in previous studies (22, 32, 47, 49–51). For IHC, an anti-MmuPV1 E4 rabbit polyclonal antibody (a generous gift from Dr. John Doorbar’s laboratory), anti-MmuPV1 L1 monoclonal antibody (MPV.B9), Ki67 (Invitrogen, rabbit monoclonal antibody, MA5-14520), and CD31 (PECAM-1) (D8V9E) XP Rabbit mAb #77699 (Cell Signaling) were used on formalin-fixed paraffin-embedded (FFPE) sections (52).

RNA ISH was performed on FFPE mouse tissue sections using RNAscope probes (the probe targeting MmPV1 E4 transcript was used in the current study), and protocols (Advanced Cell Diagnostics, ACD) as shown in a previous study (49). The HybEZ hybridization system of ACD was used to perform RNA-ISH. In brief, 5 µm sections were baked in the HybEZ Oven II (Advanced Cell Diagnostics; 321720) for 1 hour at 60°C and immediately deparaffinized in xylene, then rehydrated in an ethanol series. Epitope retrieval was performed by placing the slides in RNAscope 1 × Target Retrieval Reagent (Advanced Cell Diagnostics; 322000) at 100°C for 15 minutes and then washing it in 1 × washing buffer. Protease treatment was performed by adding RNAscope Protease Plus (Advanced Cell Diagnostics; 322331) to the section and incubating for 30 minutes at 40°C in a HybEZ Oven II (Advanced Cell Diagnostics; 321720). After probe hybridization with target probes, preamplifier, and amplifier, sections were stained with Fat Brown reagent (RNAscope 2.5 HD Detection Reagents—Brown; Advanced Cell Diagnostics; 322360). A counterstain of 50% hematoxylin and 0.02% ammonia water was used. Positive and negative probes were used in each assay to ensure proper controls. Slides from normal and infected tissues were used as negative and positive controls. Images of infected tissues with RNA ISH brown signals were captured through scanned slides using the ImageScope x64 software. Representative areas of each slide were imaged at 10× to 400× magnification and positive RNA ISH signals were counted with the specific infection status of the cells and tissues (51). E4 RNA signals should be absent in uninfected cells/tissues.

### Transmission electron microscopy (TEM)

For TEM, the tissue or pellet of the swab sample was immersion fixed for 24 h in Karnovsky’s fixative (2.5% glutaraldehyde/2% paraformaldehyde in 0.1 M sodium cacodylate buffer, pH 7.3) (32). Following fixation, the tissue was washed in 0.1 M sodium cacodylate buffer, post-fixed in buffered 1% osmium tetroxide/1.5% potassium ferrocyanide, rewashed with buffer, dehydrated in a graded series of ethanol, transferred to acetone, and embedded in Spurr low viscosity resin. Sections (70 nm) were cut with a diamond knife, mounted on copper grids, and stained with 2% aqueous uranyl acetate and lead citrate. The sections were examined in a JEOL JEM 1400 electron microscope and the pictures were recorded (32, 33).

## RESULTS

### Viral presence and anti-viral antibody determination

**Fig 2:**
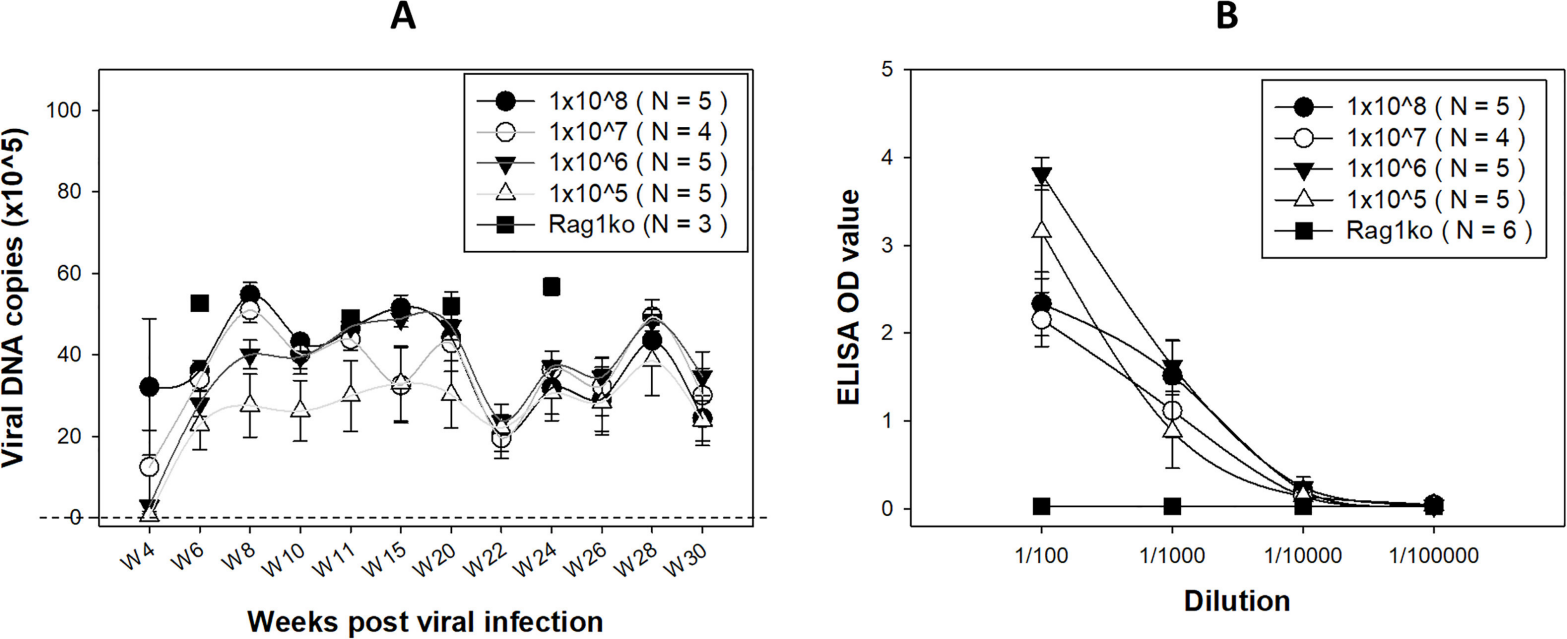
Viral DNA (**A**) over the time course of papillomavirus infection and anti-MmuPV1 E4 antibodies (**B**) were detected in the NU/J heterozygous mice. In addition to the previously used high titers of virus for infection, we also assessed a viral titer as low as 1 × 10^5^ genome equivalents. Despite a delayed detection of viral DNA copies in the group with the lower titer, no significant difference was observed among these different groups at week 22 post-infection (the dotted line indicates the threshold of viral detection). Higher levels of viral DNA were detected in Rag1ko mice at several representative time points (indicated by a square). Significantly higher levels of anti-MmuPV1 E4 IgG3 antibody were detected in all infected NU/J heterozygous mice initiated with different doses of viral infection, compared to those in Rag1ko mice (indicated by a square), at dilutions ranging from 1:100 to 1:1,000 (B, *P* < 0.05, one-way ANOVA).

In addition to six Rag1ko mice that were monitored for viral activities biweekly using our previously described method, we also conducted a dose curve study to monitor viral DNA in the NU/J heterozygous mice that we demonstrated to develop carcinoma *in situ* in a previous study (21). Consistent with previous studies (21), viral DNA levels in cervicovaginal swabs of the NU/J heterozygous mice increased and peaked around 8–10 weeks and plateaued with a drop between weeks 20 and 22 (Fig. 2A). Our previous study also observed a fluctuation in viral DNA detection that corresponded to the estrous cycle in athymic nude mice (32). Significantly higher levels of viral DNA were detected in the infected Rag1ko mice at various representative time points (Fig. 2A, square). Mice with the lowest doses of viral infection (1 × 10^5^ viral genomic copies) showed slightly lower levels of viral DNA detection by week 20 post-infection when compared to higher dose groups but no difference after week 22. Interestingly, a drop in viral DNA copies (down to 1 × 10^6^ copies/swab) was detected in all groups at week 22 post-infection (53) although these levels are about the threshold of viral DNA detection (A, the dotted line). This decrease in viral DNA detection is inversely correlated with the progression of the disease to SCC. This may suggest a potential increase in viral integration, as previously reported in MmuPV1-infected tissues (53).

NU/J heterozygous mice are immunocompetent (21). We therefore wanted to examine whether an anti-MmuPV1 antibody was generated after infection. Sera were harvested from all tested mice sacrificed at different time points up to week 30 post-viral infection. We recently reported that orally infected immunocompetent female mice develop antibodies against one of the most abundant viral proteins, E4, especially isotype IgG3 (27). Therefore, we tested these mice for E4 IgG3 isotype antibody. We ran a serial 1:10 dilution of serum samples for this assay. All infected NU/J heterozygous mice were positive for E4 isotype IgG3 despite the initial viral dose suggesting low dose such as 1 × 10^5^ is sufficient for infection in the lower genital tract (Fig. 2B) (27). Significantly higher levels of anti-MmuPV1 E4 IgG3 antibody were detected in all infected NU/J heterozygous mice initiated with different doses of viral infection, compared to those in Rag1ko mice (indicated by a square), at dilutions ranging from 1:100 to 1:1,000 (B, *P* < 0.05, one-way ANOVA) (27, 40). This agrees with previous observations that a small amount of virus is needed to initiate a productive infection either *via* blood (47) or cutaneous (54) infections.

### Virus-infected cells were identified from the cervicovaginal swabs

**Fig 3:**
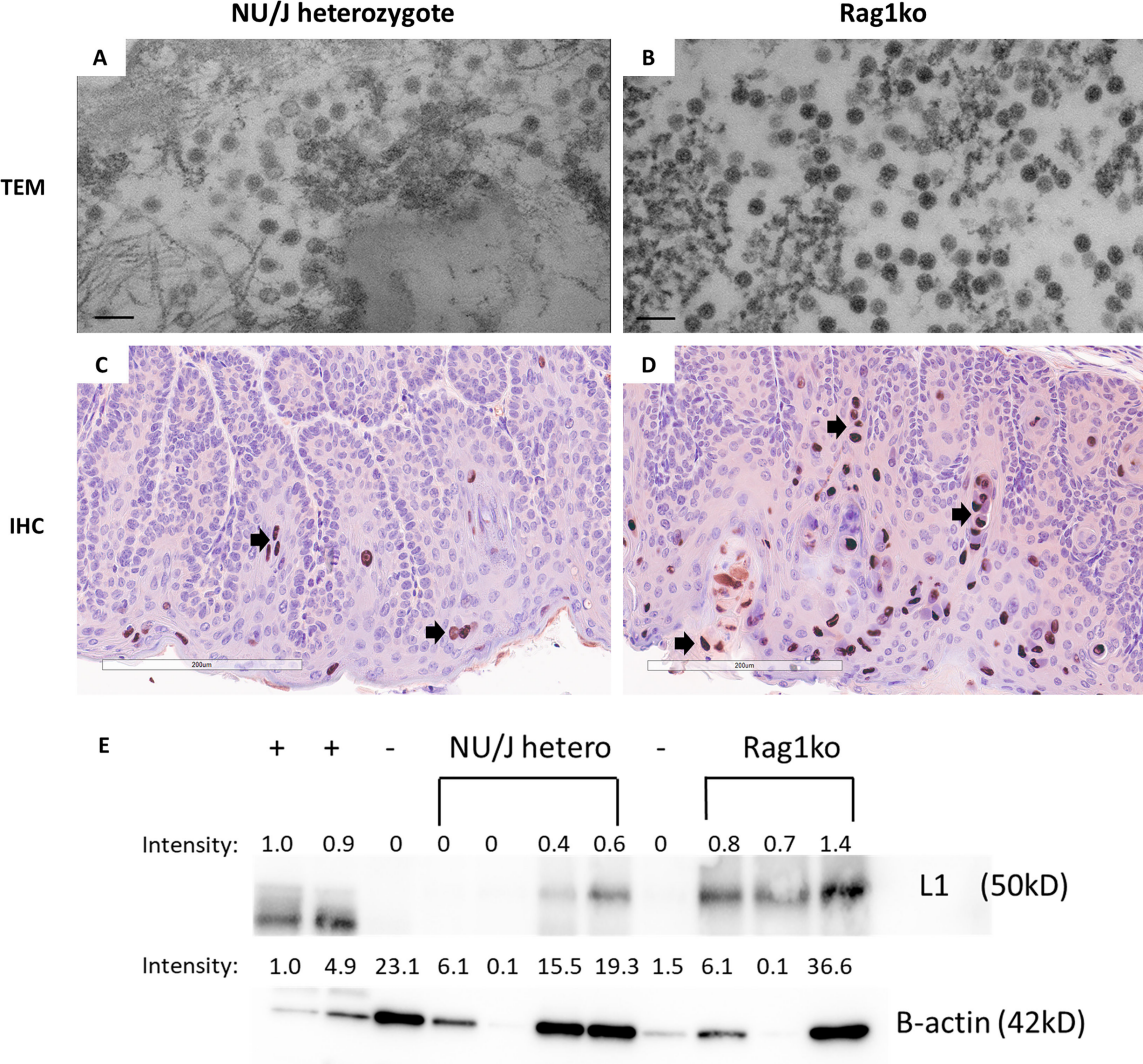
Viral particles were found in the cervicovaginal swabs of infected mice. Cervicovaginal swabs were collected from both NU/J heterozygous (**A**) and Rag1ko (**B**) infected mice and tested for viral particle presence by TEM (scale bar 100 nm). Consistent with previous reports, viral particles around 50 nm in diameter were found in both mouse strains. Rag1ko (**B**) mice produced more viral particles in the cervicovaginal swab samples when compared to those in NU/J heterozygous mice (**A**). Higher numbers of viral capsid protein L1 were detected in the infected vaginal tissues of Rag1ko (20×, **D**) mice than those in NU/J heterozygous (**C**) mice by IHC using in-house monoclonal antibody MPV.B9 (20×, Red dots, arrows). MmuPV1 L1 was detected using an in-house monoclonal antibody MPV.B9 *via* western blot (**E**).

Our previous studies have demonstrated the presence of viral particles in virus-infected tissues by transmission electron microscope (TEM) in athymic nude mice (32). To test whether virions could be detected in the exfoliated cells from the lower genital tract from our tested animals, we pooled cervicovaginal swabs from either infected Rag1ko or NU/J heterozygous mice and pelleted cells for TEM-base viral particle evaluation (Fig. 3). Consistent with previous studies, viral particles (about 50 nm in diameter) were found in exfoliated cells in the cervicovaginal samples of NU/J heterozygous (Fig. 3A) and Rag1ko (Fig. 3B) mice. The infected tissues were further analyzed for viral capsid protein L1 by IHC. In agreement with the TEM findings, viral L1 capsid protein was also detected in the infected vaginal tissues of both NU/J heterozygous (Fig. 3C) and Rag1ko (Fig. 3D) mice. The levels of MmuPV1 L1 in the infected tissues were semi-quantified by Western blot, with increased levels of L1 detected in Rag1ko mice (Fig. 3E). These findings further demonstrated that immunodeficient mice (Rag1ko) produced higher levels of viral production compared to mice with more complete immunity (NU/J heterozygotes), as reported in our previous studies (21, 51).

### Pap smear staining reveals pathogenic changes over the infection course

**Fig 4:**
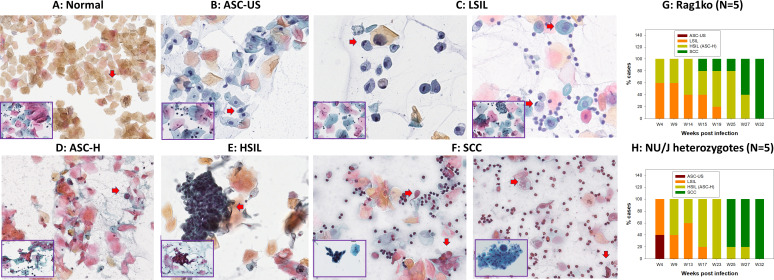
Papillomavirus infection induces stages of neoplastic progression identifiable by Papanicolaou-stained Pap smear in the form of cytologically characterized neoplasia in exfoliated cells of mouse and corresponding human Pap Smear samples (inserts at the left corner, 40×). Representative cytology images showing stages of progression of mucosal neoplasia after mouse papillomavirus infection in both immunocompromised (Rag1ko, *N* = 5) and immunocompetent (NU/J heterozygous, *N* = 5) mice infected with 1 × 10^9^ MmuPV1 viral DNA equivalent and followed longitudinally. (**A**) Normal: normal squamous cells with oval-shaped and small nuclei are among a large number of anucleate cornified squamous cells. They are negative for intraepithelial lesion or malignancy (Fig. 4A); (**B**) ASC-US: Atypical squamous cells of uncertain significance with nucleus size 1.5–2 times larger than a normal squamous cell (Fig. 4B, arrow); (**C**) LSIL: Low-grade squamous intraepithelial lesion with koilocytes (hyperchromatic raisin-shaped nuclei with nucleus size 2.5–3 times larger than a normal squamous cell and a clear halo around it, arrow; or binucleated cells with a clear halo, arrow); (**D**) ASC-H: Atypical squamous cells with hyperchromatic nucleus sized 2–2.5 times larger than a normal squamous cell and increased N-C ratio (arrow), cannot exclude HSIL; (**E**) HSIL: High-grade squamous intraepithelial lesion (HSIL, hyperchromatic cells with high N-C ratio scattered individually or forming sheets, arrow), encompassing cervical intraepithelial neoplasia grades 2 and 3; CIN2 and CIN3; and F) SCC: squamous cell carcinoma (SCC, keratinizing with marked pleomorphism of cell size and shape, arrow; the presence of tadpole cells with dense orangeophilic cytoplasm and hyperchromatic nuclei, arrow; spindle-shaped cells, and inflammation and necrotic debris on the background). These stages of neoplastic progression are based on the 2014 Bethesda system and represent increased grades of intraepithelial neoplasia. Both immunocompromised (G, Rag1ko) and immunocompetent (H, NU/J heterozygous) mice infected with MmuPV1 at the lower genital tract developed LSIL as early as week four post-infection. An increased proportion of Pap smear samples became HSIL over time in both mouse strains. After 6 months, most infected animals developed HSIL and all of them progressed to SCC after 8 months post-infection in the vaginal tissues.

Our previous studies demonstrated that virally infected abnormal cells can be determined by cytological analysis from cervicovaginal lavage samples in vaginally infected mice using H&E staining (21, 22, 32). To further determine whether Pap smear staining could reveal similar cytological changes in the longitudinal cervicovaginal swabs as reported in humans and to determine the suitability of this approach as a model for human studies, we adapted the clinical Pap smear staining protocol for our collected slides. Slides were prepared according to the protocol described in Fig. 1 from five Rag1 and five NU/J heterozygous mice infected with 1 × 10^9^ MmuPV1 viral DNA equivalent as shown in Fig. 4G and H, respectively. More than 200 Pap smear slides from infected and noninfected mice were evaluated without knowledge of infection status or time post-infection before matching the assessments with the animal ID (Table 1). In agreement with our previous cytological observations using H&E staining, we detected amphophilic cytoplasm (resembling inclusion) in atypical squamous cells with ribbon-like central chromatin (22, 32). Koilocytosis, a typic morphology of papillomavirus-infected cells, was easily identified in the slides harvested from these infected mice. The corresponding grades of the longitudinal samples were determined based on squamous cell morphology using the 2014 Bethesda system, the standard used to evaluate human Pap smears (Fig. 4A through F). Here, we showed an example of each category. We found a high convergence between mouse and human Pap smear slide diagnoses (insets of Fig. 4A through F). Increased HSIL cases were observed in both longitudinally Pap smear samples of Rag1ko (*N* = 5, Fig. 4G) and NU/J heterozygous (*N* = 5, Fig. 4H) mice after 6 months post-infection. All Rag1ko and NU/J heterozygous infected mice developed SCC according to cytological characterization after 8 months post-infection for both mouse strains.

### Validation of disease progression by *in situ* assays

**Fig 5:**
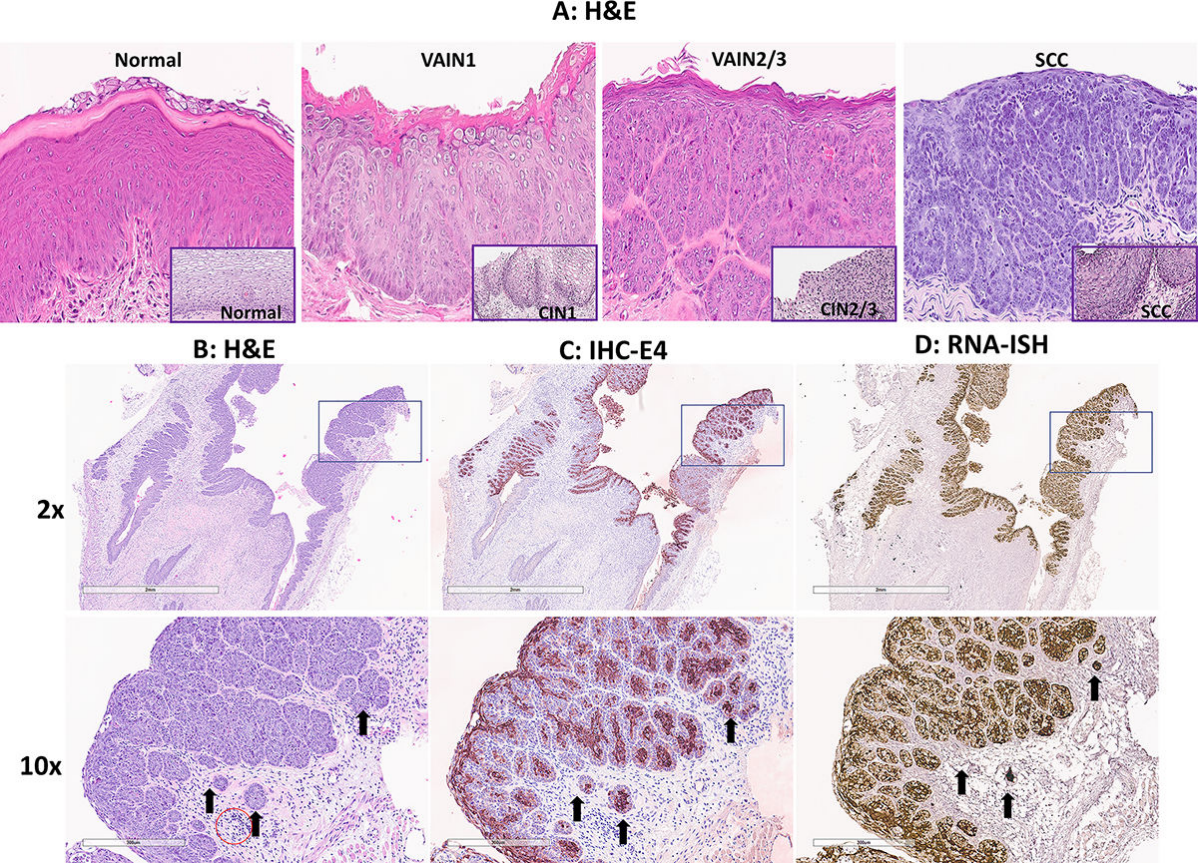
Papillomavirus infection induces identifiable stages from vaginal intraepithelial neoplasia (VAIN) to squamous cell carcinoma (**A**). Infected mice were sacrificed at different time points post-viral infection for histological validation. We observed the agreement between Pap smear LSIL and VAIN1; HSIL and VAIN2/3; and SCC in our tested animals. Overall, it may take around 5.5 months after mouse papillomavirus infection in the mucosa of infected mice to develop advanced diseases (HSIL to SCC). All mice developed SCC after 8 months post-infection. Corresponding human cervical intraepithelial neoplasia tissues (A, inserts at the right corner, 20×) share similar pathology with the mouse tissues. Histology of squamous cell carcinoma (2× and 10×, **B**) with a scirrhous response (B, arrows) typical of carcinomas was detected in SCC. Strong viral E4 protein (2× and 10×, C, arrows) and RNA transcripts (2× and 10×, D, arrows) were detected by IHC and RNA-ISH, respectively. Most inflammation was lymphoplasmacytic. There is a low level of immune cells in the stroma of the vagina (red circle), but this cluster was more than anticipated = score 1.

To determine whether cytological results agreed with the histology of infected mice, we sacrificed Rag1ko and NU/J heterozygous mice at different time points post-viral infection. Viral signals, including viral DNA, RNA, and proteins, were detected as previously reported (21, 51). The histology of infected vaginal tissues of both mouse strains agreed with the Pap smear samples on the disease stages. We detected vaginal intraepithelial neoplasia (VAIN) 1 to squamous cell carcinoma (SCC) in the vaginal tissues of these infected mice over time (Fig. 5A). Surprisingly, we also detected SCC in NU/J heterozygous mice as early as 21 weeks post-virus infection in the current study. After week 28 post-infection, most tested mice (both Rag1ko and NU/J heterozygous mice) developed SCC at the infected vaginal tissues (Fig. 5B). The histological changes agree with HPV-induced cervical intraepithelial neoplasia shown in the corresponding insert of Fig. 5A.

NU/J heterozygous mice were susceptible to MmuPV1 infection and developed VAIN3 in our previous study (21). In the current study, we further identified SCC in these infected mice. The histology of the infected tissues agreed with the Pap smear diagnoses collected simultaneously. In other words, the infected tissues of Pap smear SCC samples also displayed SCC by histological characterization. Figure 4B (2 × and 10×) shows an example of a scirrhous response typical of carcinomas of vaginal SCC tissues of a NU/J heterozygous mouse. The SCC tissues were further evaluated for viral presence using antibodies and probes targeting MmuPV1 E4 (Fig. 5C), an abundant protein produced after active viral infection (32). These SCC tissues are also positive for MmuPV1 E4 RNA transcripts (Fig. 5D). The infected cells invaded the underlying vaginal lamina propria and muscularis layers (Fig. 5B through D, arrows). Most inflammation was lymphoplasmacytic as shown in Fig. 5B. There is a low level of immune cells in the stroma of the vagina (Fig. 5B, red circle) but this cluster was more than anticipated = score 1.

### Increased Ki67 and CD31 expression in the infected vaginal tissue that developed SCC

**Fig 6:**
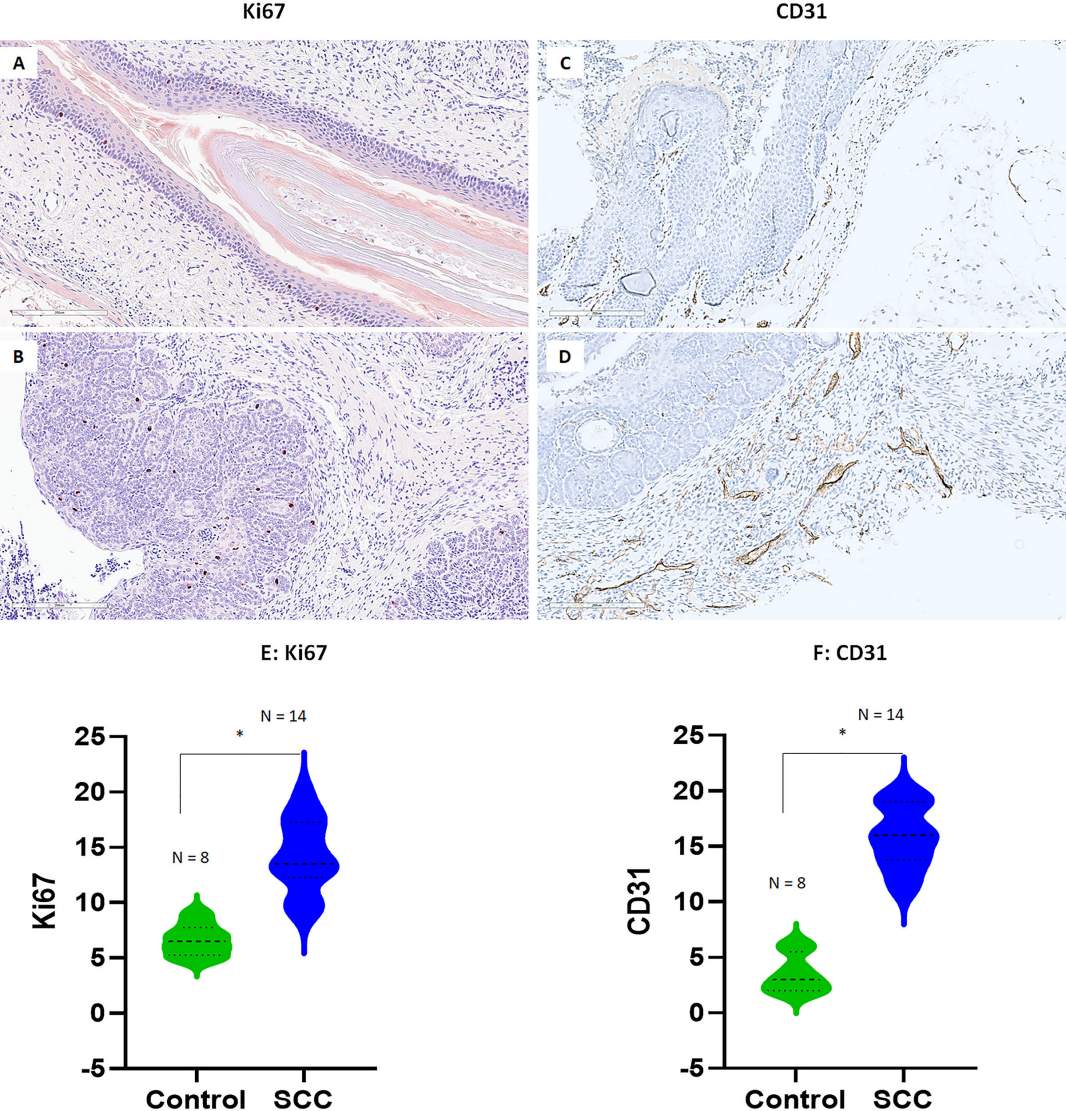
Increased mitotic signal (Ki67) and endothelial cellular adhesion molecule (CD31) were found in representative infected mucosa of mice. Representative images of either Ki67 or CD31 expression in the normal (20×, **A, C**) or infected vaginal tissues with advanced SCC (20×, **B, D**) of both tested Rag1ko and NU/J heterozygous mice. Significantly higher Ki67-positive cells (E, *P* < 0.01, unpaired Student’s *t-*test) and CD31-positive blood vessels (F, *P* < 0.01, unpaired Student’s *t*-test) were found in SCC tissues when compared to those in normal tissues.

Several biomarkers have been widely used for HPV pathogenesis and SCC (52, 55, 56). For example, cellular proliferation with increased numbers of mitotic figures in the nuclei could be found by Ki67 staining in tissues with more advanced diseases including SCC when compared to that of the normal tissues (20, 57). In addition, among many molecules involved in tumor angiogenesis, platelet-endothelial cellular adhesion molecule (PECAM-1), also known as CD31, has been widely used as a biomarker in highlighting tumor vascular invasion (52). Increased vascular growth has been found in HPV-infected tissues with advanced diseases and cancer and can be targeted for therapy (52, 58). We therefore conducted immunohistochemistry analysis to probe the changes in Ki67 (nuclear staining) and CD31 (vascular staining) expression in the vaginal tissue with normal (Fig. 6A) or low-grade disease (Fig. 6C) and SCC (Fig. 6B and D). For Ki67, we focused on the signals in the infected regions and compared them to corresponding control tissues. Ki67 was found in the nucleus of basal epithelial cells of both normal and infected cells that were actively growing and dividing. Based on the criteria used for Ki67 staining used in clinical evaluation (55), we counted Ki67-positive cell numbers of a 20× magnification view of both normal (*N* = 8) and infected tissues (*N* = 14). Although we detected Ki67-positive cells in both normal (Fig. 6A) and SCC (Fig. 6B) tissues, significantly higher numbers of Ki67-positive cells were found in SCC tissues (E, *P* < 0.01, unpaired Student’s *t*-test). Similarly, we counted CD31-positive signals of a 20× magnification view based on the size of vascular staining. Significantly higher levels of CD31-positive blood vessels were found in SCC (Fig. 6D) when compared to those in normal controls (Fig. 6F, *P* < 0.01, unpaired Student’s *t*-test) supporting the role of CD31 in tumor growth and malignancies.

## DISCUSSION

To the best of our knowledge, this is the first report on longitudinally following viral infection to squamous cell carcinoma stages using the well-established mouse model to recapitulate human HPV lower genital tract infection and dysplasia. This study suggests that Pap smear together with viral DNA detection effectively monitored disease progression in the mouse model. These findings will allow us to better understand the dynamic changes during tumor progression in the lower genital tract and develop and test novel treatments to counteract disease progression at different disease stages.

As the fourth most common cancer, cervical cancer not only claims the lives of more than 300,000 annually but has become a heavy economic and health burden worldwide (6). In the United States, around 5.5 million new HPV infections are reported each year. Among them, 10% of HPV persistent infections progress to invasive cancers, which can take more than two decades (59). Treating HPV-associated low-grade and high-grade squamous intraepithelial lesions (SILs) costs billions of dollars each year in the United States (60). Therefore, novel strategies to prevent and treat HPV-associated diseases and cancers besides the HPV vaccines are highly desired.

Since the first report of the MmuPV1 as a cutaneous virus (61), this model has been studied extensively and helped to answer many questions relating to papillomavirus infection and pathogenesis (18, 34, 62, 63). For example, we use this model to understand infection at different anatomic sites and how host immunogenetic background contributes to different disease outcomes (19, 24, 40, 47). These studies will help us further answer some clinical questions including the role of both viral and host genes and pathways in infection control and discover novel biomarkers for diagnosis and therapies (34, 62, 63). MmuPV1 is a member of the pi papillomavirus genera and thus evolutionarily distinct from the alpha papillomaviruses most often associated with human cancers (64). However, MmuPV1 contains two functional oncogenes, E6 and E7, and a non-coding region with many of the same regulatory and transcription factor binding sites as shown in HPV genomes (34, 65) and most importantly, a tissue tropism that includes the anogenital tract (33, 39). The broad tissue tropism of the MmuPV1 has allowed us to model several types of prevalent HPV infections and cancers including oropharyngeal cancer in this amiable laboratory animal model (33, 66–68). Over the past decade, we have had a better understanding of viral-host interaction at both cutaneous and mucosal sites using this model system with the collective efforts of many researchers (19, 20, 23–31, 34, 38–40, 47, 49, 51, 53, 54, 62–65, 69–83). The most promising advancement is identifying additional immunocompetent mouse strains for studying viral persistence and clearance in different tissue types (19–22, 38, 70, 73). This tractable model is highly desirable especially for mucosal infections, as we can monitor disease progression longitudinally without sacrificing animals for the prospective study of HPV pathogenesis (22, 32).

Our previous cytological assay used H&E stain to determine abnormal cells in our infected animals (22, 32). To facilitate the clinical application of this model to discover mechanisms underlying disease progression and develop novel diagnostic and therapeutic markers, we adapted the well-established Pap smear protocol for human samples. To further determine whether Pap smear staining could reveal similar cytological changes in the longitudinal cervicovaginal swab samples as reported in humans, two pathologists independently evaluated more than 200 slides from infected and noninfected mice without knowledge of infection status or time post-infection before matching the assessments with the animal ID. There was high agreement between pathologists, suggesting the translated Bethesda scoring system and evaluation are consistent and robust for evaluating the Pap smear slides from mice. The discrepancies between the two diagnoses in some samples represent the natural spectrum of pathological evaluation in humans. We were able to monitor changes and correlate disease stages using Pap smear with corresponding human specimens. Therefore, the Pap smear is a valid and efficient method for monitoring MmuPV1-associated cervicovaginal disease progression longitudinally. We detected fluctuation in viral DNA detection according to the estrous state in the genital-infected athymic mice (32). In the current study, we attempted to correlate the estrus stage with Pap smear evaluation. However, it was impossible to identify the estrous state from virus-infected mouse Pap smear samples, especially later, due to increased signs of infection secondary to neoplastic invasion and cellular atypia. Therefore, we did not classify Pap smear samples by stage of estrous cycle in the infected slides.

We used Rag1ko mice and NU/J heterozygotes to represent both immunocompromised and immunocompetent backgrounds, respectively. Similar genetic deficiencies were found in humans (41, 84). Therefore, our mouse model can potentially be used to study the interaction between viral and host during papillomavirus infection in these patients which has not been reported. The primary and secondary immunocompromised people are more prone to HPV infections and associated malignancies (85). To generate robust data for Pap smear to cover all the cytological characterization stages, we followed both immunocompromised (Rag1ko) and immunocompetent (NU/J heterozygous) mice up to 32 weeks post-infection. Biweekly swabs were evaluated for both viral load and cytology. In addition, corresponding tissues were harvested periodically along with cytological samples for further validation. This design allowed us to gain time-sensitive data to closely monitor disease progression in these infected animals. Although Pap smears in human studies focus on cervical cytology, cervicovaginal swabs have been reported to screen for HPV infections in women using self-sampling for different populations and showed satisfactory results (86–88). The self-sampling strategy to collect cervix-vaginal swabs for HPV detection can overcome Pap smear challenges, including the requirement of professionals to collect Pap smear samples and the cost of conducting the procedure but cannot detect cytological changes (86). A recent study demonstrated that a combination of cytology and HPV testing would improve the detection of early cases of CIN2+ (89). In our study, we also combined viral detection, cytology, and tissue histology for more accurate evaluation of these infected tissues as used in clinical practice. The agreement between our mouse model and reports in humans further supports that this combination is essential for early diagnosis and development of novel stage-associated treatments for HPV-associated cancers.

Intriguingly, malignant conversion was detected in both immunocompromised (Rag1ko) and immunocompetent (NU/J heterozygous) mice as early as 22 weeks after viral infection in Pap smears and the corresponding tissues. Rag1ko mice are deficient in both T and B cells, key components of the adaptive immune system; therefore, MmuPV1 infection at both cutaneous and mucosal tissue persists and develops cancer over time. All tested immunocompetent NU/J heterozygous mice also developed SCC in the vaginal tissues in the current study. Our previous study also detected VAIN3 in NU/J heterozygous mice (21). Advanced disease including SCC in the infected genital tract was also reported in another immunocompetent mouse strain (FVB) after 6 months of infection (20) and some immune-deficient mouse strains by other groups (19). The mechanisms underlying SCC development in mice from different genetic backgrounds need further investigation. Ki67- (a cellular marker for proliferation) positive cells are found in high-risk HPV-associated cancer (55). Aligned with this, we also observed significantly higher numbers of Ki67-positive basal cells in infected epithelia of NU/J heterozygous mice compared to controls as shown in the infected FVB mice (20). However, more Ki67 signals were shown in the infected FVB vaginal tract in a previous study (20). The main difference was the type of the antibody for Ki67. We used a rabbit monoclonal antibody that usually shows a lower background compared to a mouse monoclonal antibody on mouse tissues. To confirm our observations, we tested infected FVB mice using our rabbit monoclonal antibody and detected a similar pattern as shown in NU/J heterozygous mice suggesting the different Ki67 antibodies contributed to these differences (Fig. S1A through C). Other factors were also reported to impact the detection of Ki67 in the vaginal tissues, these include hormone levels and estrus stages (90–92). The previous study treated FVB mice with 17-beta estradiol (20) while no hormone treatment was applied to either FVB or NU/J heterozygotes after the initial DMPA treatment for standard infection in our study. Whether 17-beta estradiol contributed to increased Ki67 detection in FVB mice needs further investigation. Nevertheless, we both observed the same increase in Ki67-positive cells in the infected basal layers compared to uninfected tissues. CD31 is a reliable marker of tumor vascular invasion and HPV malignancies. We further demonstrate the correlation between increased expression and advanced disease stages in both NU/J heterozygous and Rag1ko mice suggesting it can be a tumor invasion marker for cancer in the mouse model. Therefore, the mouse model recapitulates HPV-associated disease progression from early infection to SCC, making it a relevant model to study HPV pathogenesis. In our previous study, we observed increased Ly6B.2-positive cells in the lower genital tract of NU/J heterozygous mice with advanced disease (21). In the current Pap smear samples, we also detected increased numbers of neutrophils in advanced stages especially HSIL samples suggesting these immune cells might have played a role in accelerating cancer development. Infiltration of Ly6B.2-positive cells were found in tissues with high-grade dysplasia (21). We showed most heterozygous mice (9/10) showed increased neutrophil infiltration while only one of five Rag1ko mice showed neutrophil infiltration in the infected vaginal tissues (Fig. S1D through F) (21). Further investigation needs to be conducted to determine their function in HPV pathogenesis.

Both cytology and histology agree with each other in our tested animals, especially at the advanced disease stage such as HSIL and SCC. For some samples, HSIL was detected as early as 8 weeks after infection but fluctuated until three continuous time points of HSIL to correlate with SCC development in these mice. SCC was detected as early as 16 weeks post-infection in some mice. Tissues harvested at the corresponding time points also showed similar pathogenic stages, suggesting that Pap smear-based cytological evaluation is valid for diagnosing the disease progression. It is especially significant that we can use this traceable model to test anti-viral and anti-tumor compounds by intervening at different time points/disease stages and to differentiate their efficacy in clearing infections/tumors.

## Data Availability

The data supporting the findings of this study are available from the corresponding author upon request.

## SUPPLEMENTAL MATERIAL

Figure S1 mbio.01420-24-s0001.docx Increased mitotic signal (Ki67) and neutrophils (Ly6B.2) in red were found in vaginal tissues by immunohistochemistry.

ASM does not own the copyrights to Supplemental Material that may be linked to, or accessed through, an article. The authors have granted ASM a non-exclusive, world-wide license to publish the Supplemental Material files. Please contact the corresponding author directly for reuse.
