# Supplementary material for: Monitoring mouse papillomavirus-associated cancer development using longitudinal Pap smear screening
Source: mBio. 2024 Jul 16;15(8):e01420-24. doi: 10.1128/mbio.01420-24 (PMC11323795; doi:10.1128/mbio.01420-24)
Supplement: Figure S1 — Increased mitotic signal (Ki67) and neutrophils (Ly6B.2) in red were found in vaginal tissues by immunohistochemistry. [file mbio.01420-24-s0001.docx]

**Supplementary Figure S1.** Increased mitotic signal (Ki67) and neutrophils (Ly6B.2) in red were found in vaginal tissues by immunohistochemistry. No Ki67 expression was found in most normal vaginal tissues (20×, A) but one with similar pattern at the basal layers as shown in Spurgeon 2019 mBio (20×, arrows, B). Ki67 expression in FVB mice infected with MmuPV1 was tested using our rabbit monoclonal antibody. We also observed a similar pattern and levels of Ki67 signals as we found in NU/J heterozygous mice (20×, arrows, C). While most Rag1ko (5/6) mice showed minimal signals (20×, D) except one (20×, arrows, E), majority infected vaginal tissues of NU/J heterozygous mice (9/10) showed neutrophils infiltration (20×, arrows, F) as reported previously.


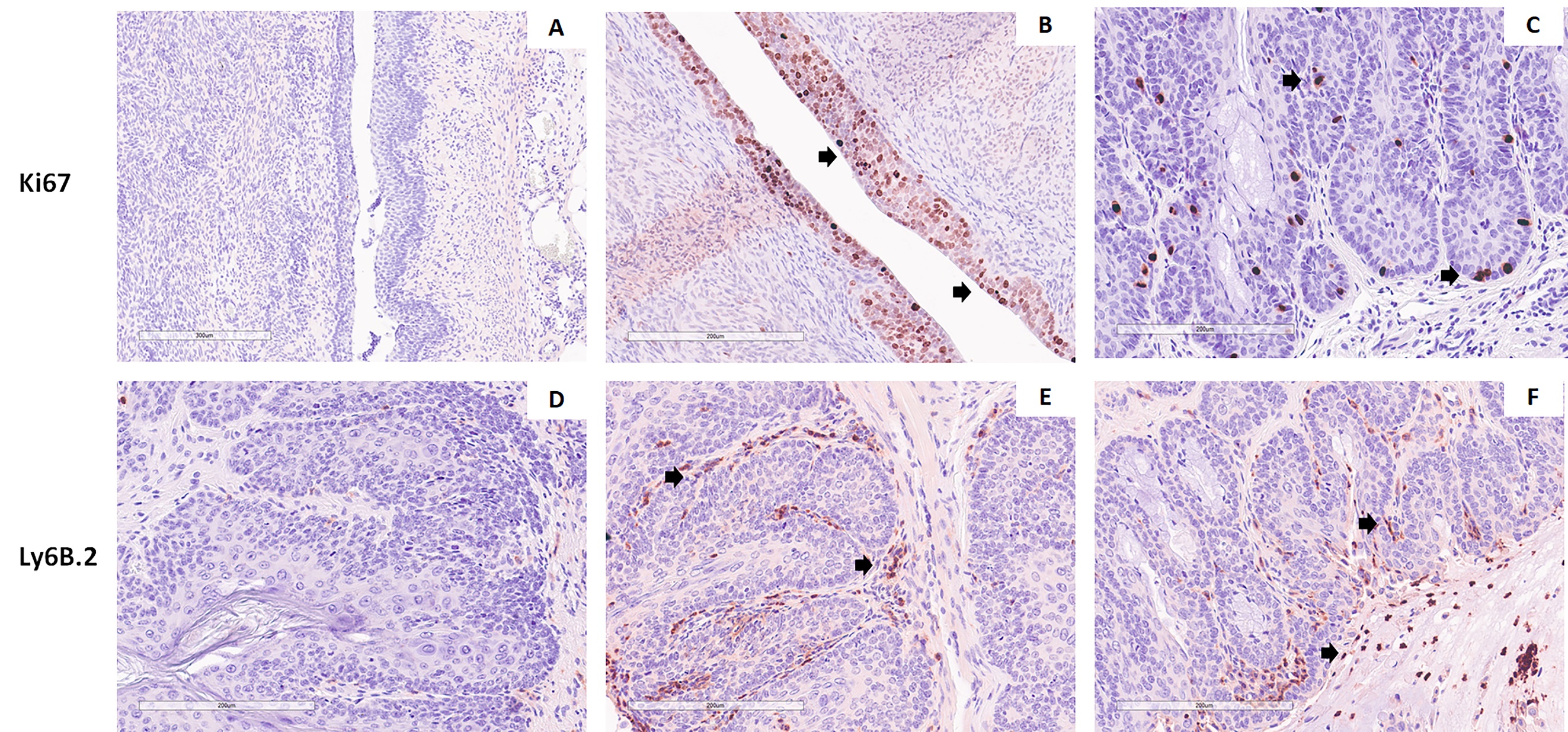


**Supplementary Fig 1. Increased mitotic signal (Ki67) and neutrophils (Ly6B.2) in red were found in vaginal tissues by immunohistochemistry.** No Ki67 expression was found in most normal vaginal tissues (20×, A) but one with similar pattern at the basal layers as shown in Spurgeon 2019 mBio (20×, arrows, B). Ki67 expression in FVB mice infected with MmuPV1 was tested using our rabbit monoclonal antibody. We also observed a similar pattern and levels of Ki67 signals as we found in NU/J heterozygous mice (20×, arrows, C). While most Rag1ko (5/6) mice showed minimal signals (20×, D) except one (20×, arrows, E), majority infected vaginal tissues of NU/J heterozygous mice (9/10) showed Ly6B.2 positive cell infiltration (20×, arrows, F) as reported previously.
